# Identification of Biomechanical Properties of Temporomandibular Discs

**DOI:** 10.1155/2020/6032832

**Published:** 2020-10-07

**Authors:** Edward Kijak, Jerzy Margielewicz, Małgorzata Pihut

**Affiliations:** ^1^Department of Prosthetic Dentistry, Faculty of Medicine and Dentistry, Pomeranian Medical University, 1 Rybacka, Szczecin 70-204, Poland; ^2^Department of Prosthetic Dentistry, Faculty of Medicine and Dentistry, Wroclaw Medical University, 1 Ludwika Pasteura, Wrocław 50-367, Poland; ^3^Silesian University of Technology, Faculty of Transport and Aviation Engineering, 8 Krasińskiego, Katowice 40-019, Poland; ^4^Department of Prosthetic Dentistry, Jagiellonian University Medical College, Montelupich, Krakow 31-155, Poland

## Abstract

**Material:**

Experimental and model tests were conducted on ten fresh porcine temporomandibular joint discs. The average thickness of disc tissue was, accordingly, 2.77 mm for the anterior zone, 3.98 mm for the posterior, and 1.54 mm for the intermediate. The selection of research material in the form of porcine discs was due to the similarity to human discs.

**Methods:**

Discs were loaded in cycles, a temporary course with the amplitude 3 N and frequency 0.07 Hz, and growth in the load was 1 N/s. The selection of load frequency was due to real conditions of temporomandibular joint functioning during mastication. The necessary experimental research was conducted on a testing machine with a measurement range of 2.5 kN.

**Results:**

The obtained numeric calculation results indicate that the number of load cycles has a decisive impact on the limitation of energy dispersion capacity through disc tissue. This phenomenon was observed in all the studies on the disc areas. Along with the growth in load cycles, discs are stiffened, and the most significant stiffness was observed in the intermediate area.

**Conclusions:**

Based on the conducted research, it should be concluded that excessive load affecting temporomandibular joints caused by the act of mastication and occlusal forces generated during parafunction and in people with defined long-term bruxism has crucial importance on biomechanical disc properties and hence the course of temporomandibular joint conditions.

## 1. Introduction

Temporomandibular disorder (TMD) is an umbrella term including group of conditions that cause pain and dysfunction in the masticatory muscles, the temporomandibular joint (TMJ), and their associated structures. They are a group of complaints associated with accelerating life pace, and therefore, we observe an increase in the incidence of this type of disorders. Stress, fatigue, anxiety, depression, sleep disorders, and a fast pace of life affect the human psyche [1] negatively. TMD and oral parafunctions seem to be a frequent problem in modern societies [2–5]. The etiopathology of the TMD is related to teeth arches, muscles, and periodontium. Their main causes involve both pathophysiological and psychosocial factors [6, 7]. The primary symptoms include pain during mandible movements, limitation of mandibular mobility, and acoustic symptoms recorded within TMJs [8]. It is estimated that TMD may relate from 75 to 90% of the surveyed population [9–11].

Epidemiologic tests, conducted in recent years, focus on the factors causing functional and morphological changes in the stomatognathic system. It has been proved that the multifactorial etiological causes may have a biopsychosocial base, as well as occlusal disorders, stress factors, mental conditions, generalized diseases of joints and hormonal disorders, congenital defects, headaches, and arthritis [12–16]. The latest extended TMD (DC/TMD) taxonomy takes into account as many as 37 disorders considered, which have been grouped into the following four categories: temporomandibular joint disorders, chewing muscle disorders, headache disorders, and related structure disorders [17].

The most common cause of the TMD is stress, especially long-term stress [18, 19]. Some studies show that headache is a much more common problem for participants with painful TMD [20]. Furthermore, some studies underline also the gender influence on TMD development. It has been proved that being under stress increases the activity of the masticatory muscles, which consequently results in TMD [21, 22].

Sleep disorders associated with stress include bruxism. Bruxism is an oral habit consisting of involuntary rhythmic or spasmodic nonfunctional gnashing, grinding, or clenching of teeth, unlike chewing movements of the mandible, which may lead to occlusal trauma that is characterized by the clenching and grinding of teeth. It can occur as awake bruxism (AB) and sleep bruxism (SB). AB is a distinct entity from SB and is characterized mainly by the clenching of teeth [23].

The sleep and awake bruxism are generally considered as different behaviours observed during sleep and wakefulness, respectively. It is therefore more appropriate to use a definition or in principle two separate definitions, which were developed under the International Consensus in 2018. So by sleep bruxism, one should understand “a masticatory muscle activity during sleep that is characterized as rhythmic (phasic) or nonrhythmic (tonic) and is not a movement disorder or a sleep disorder in otherwise healthy individuals.” Awake bruxism is, according to the authors, “a masticatory muscle activity during wakefulness that is characterized by repetitive or sustained tooth contact and/or by bracing or thrusting of the mandible and is not a movement disorder in otherwise healthy individuals” [24]. The prevalence of AB in adults was reported to range from 22.1% to 31% while that of “frequent” SB was more consistent at 13% [25]. The bite forces during SB events can exceed the amplitude of maximum voluntary bite force when awake. The aforementioned leads to significant loading of teeth, the periodontium, temporomandibular joint (TMJ), as well as muscles of mastication [26].

An integral element of the mastication organ is the temporomandibular joints, the place of the moveable connection of the mandibular condyle with immobile cranial bones. The movements of both the mandibular condyle in the joints are coupled; i.e., despite having different natures, they proceed simultaneously. The nature of the movements of the mandibular condyle in the TMJ depends primarily on the structure of the joint, ligaments, and biomechanical properties of the discs. From a clinical point of view, the disc divides the articular cavity to the separate cavities: upper and lower, and the main role in its functioning is played by movement of fluids through the interstitial areas [27]. Cartilage tissues are sensitive to their mechanical environments [28].

The specific biomechanical properties of TMJ discs have a significant impact on the biomechanics of the temporomandibular joints. The mechanical function of the TMJ disc is determined by the composition and structure of its distinct extracellular matrix (ECM) [29].

The human TMJ disc is largely avascular, so cellular nutrients essential for maintaining a healthy ECM must be supplied by diffusion from the synovial fluid and blood vessels in the disc periphery [29]. Some studies have shown that mechanical loading limits nutrient availability within the TMJ disc by altering diffusivity. Electrical conductivity, a material property of biological tissues related to ion diffusivity in soft, hydrated tissues, decreased with increasing mechanical strain [30].

Studies conducted with the use of magnetic resonance (MR) indicate that, during movement of the mandibular condyle from the fovea articularis to the articular tubercle, discs are deformed [31]. Disc deformation is reflected by its narrowing or extension, depending on the current position of the heads in the articular space. In the work by Fung, attention was paid to the fact that the edges of TMJ discs are stiff and capable of transferring loads, while the intermediate zone is thin and flexible [32]. The main purpose of the external stiff areas of TMJ discs is to protect against an excessive increase in contact stresses between areas of heads and acetabulum [33].

The phenomena occurring in TMJ discs tissue during its deformation are difficult to direct observation. It is possible, however, to recognise them by applying model tests [34, 35], and an extremely useful piece of technique is the finite elements method [36, 37]. The compliance of the obtained computer simulation results with the state of the real structure is a necessary condition for skilful separation of the issue, by adopting appropriate boundary conditions and introducing material data typical of TMJ disc tissues properties. The purpose being an evaluation of biomechanical properties of TMJ discs, a particularly significant phase, seems to be the material data collection. Common awareness of this fact results in the fact that much attention was devoted to the evaluation of biomechanical properties of natural tissues, responsible for the transfer of forces, generated during motor activities. In the case of joints, the key problem is to define the biomechanical properties of the disc, taking into consideration diverse features of its particular fragments. The variety of material features facilitates the cooperation of mandibular condylar heads with panes of different shapes [38].

The main aim of this study was to assess the mechanical properties of pig joint discs as analogues of human TMJ disc. This identification was conducted based on recorded measurement data obtained during cyclic disc loading. The obtained data were the basis for determining the scattering and elastic properties of the joint disc. It is an attempt to mathematically describe the biomechanical properties of the articular disc tissues, in the anterior, intermediate, and posterior zones, which are subjected to cyclic loading. The results presented in this paper can be treated as a supplement to the information on specific biomechanical data, characterizing the elastic properties and the ability to dissipate energy by the tissues of the TMJ disc.

## 2. Materials and Methods

The object of identification of elastic and dissipation properties was on ten fresh porcine TMJ discs that were analyzed in three zones: the anterior (AZ), the intermediate (IZ), and the posterior (PZ). The porcine disc was chosen based on geometric, microstructural, and biochemical similarities to the human specimens. The average length in medial-lateral direction was 2.68 ± 0.214 cm, and average length in anterior-posterior direction was 1.41 ± 0.116 cm. However, the average thickness of the tissues of the disc in different studied areas amounted to the following: the anterior area 2.77 ± 0.101 mm, the posterior 3.98 ± 0.216 mm, and the intermediate 1.54 ± 0.060 mm. Research material was isolated from dead pig heads prepared within industrial slaughter meat plants. TMJ discs were obtained from a local abattoir in a manner consistent with institutional regulations. All the TMJ discs have been carefully dissected from pig heads by one experienced maxillofacial surgeon. The discs were identified and stored separately in 0.1 M phosphate buffered saline (PBS, pH = 7.2). During the experiments, the discs were kept at 37°C in PBS. All discs were identical in size because they were obtained from similar 100 kg animals. The experiments for each disc were completed during the day the test material was collected.

The first stage of the research was the definition of research material selection criteria. The fundamental requirement assumed no mechanical damage of TMJ disc tissues. The basis for conducting any model tests concerning the identification of mechanical properties of biological or technical materials is measurement data. In the presented work, laboratory tests were carried out on a universal testing machine, namely, Zwick, with a load range of 2.5 kN, controlled via the computer program Test Xpert. To exert a direct load on discs, a specially designed measurement stamp was used with a diameter of 5 mm, whose contact surface had a shape close to disc geometry. Adjusting the stamp's shape to the circumferential geometry of the disc was aimed at minimizing uncontrolled slips. After appropriate disc placement towards the stamp, it was loaded in cycles, with a saw-tooth course with constant load increase speed, amounting to 1 N/s, and a frequency equal to 0.07 Hz. Such a frequency approximately corresponds to real-functioning conditions of the temporomandibular joints during mastication. The frequency was determined based on own chewing measurement data recorded with the Zebris JMA electronic facial arch in a healthy, model patient [39]. The experimental studies do not reproduce the actual movement that takes place in the temporomandibular joints because it is so complicated that it is difficult to reproduce it on a testing machine. Nevertheless, care has been taken to ensure that the frequency with which the joint disc tissues is loaded corresponding to the actual loads during the act of chewing.

**Figure 1:**
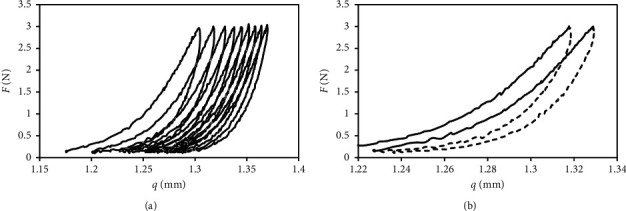
Force-displacement characteristics of TMJ discs: (a) ignoring the first load cycle; (b) separated load phases and unloading different cycles.

Additionally, a cycle from zero was implemented to the maximum value of 3 N. Border upper scope load has been adopted for the experiment in such a way so as not to cause excessive disc tissue crushing. Additionally, in the lower scope of cycles, the load remained insignificant, ensuring continuous disc contact with the measuring stamp. This negligible pressure in the lower load range assured that, in each subsequent cycle, disc tissues were loaded precisely in the same place. During the tests, we recorded force signals and displacement of the transverse beam of the testing machine synchronously. The recorded temporary runs of force signals and displacement were presented in the form of force-displacement characteristics (Figures 1(a) and 1(b)).

Because of difficulties in obtaining the repeatability of results, for model tests, only cases were selected with regular hysteresis loops, i.e., ones that did not demonstrate changes in the shape. Changes in the shape of the hysteresis loop were caused mainly by TMJ discs sliding on the measuring table and the excessive presence of soft tissue residues, left after preparing. Taking account of imprecise disc tissues preparation, biomechanical properties of the discs were identified only from the second cycle, as this cycle, in principle, may be considered stable. It is worth emphasising the fact that the impact of this type of disturbances is lower with each cycle that follows because residues in other soft tissues are being crushed. Consequently, their effect on the recorded characteristics is much limited.

**Figure 2:**
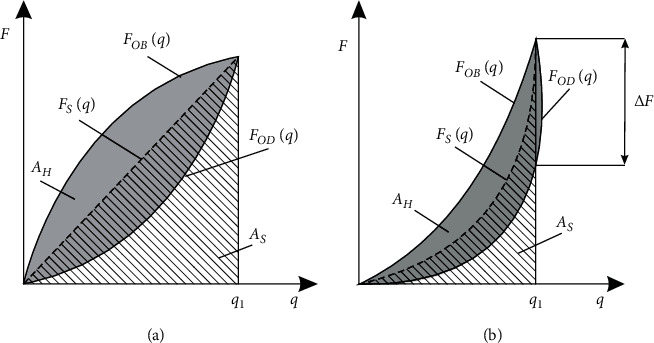
Hysteresis loops on the basis of which the nondimensional energy dispersion coefficient is identified: (a) material with linear properties; (b) material with nonlinear properties.

The first stage of identification is separation from the recorded characteristics, particular load cycles and curves, corresponding to loading and unloading phases (Figure 1(b)). The next stage covers the characteristics' shift to the beginning of the coordinate system. The activity is intended to clear one of the integration boundaries as well as the simplification of the analytical functions record. At this point, it should be pointed out that process in such a way and measurement data do not affect the recorded hysteresis loops. During the cyclical TMJ disc tissues loading, part of the mechanical energy is dispersed. This phenomenon is associated, above all, with plastic deformation, and its size in a single load cycle is defined based on the surface area of the hysteresis loop (Figures 2(a) and 2(b).

On the basis of being recorded in different loading cycles of the hysteresis loops, the energy dispersion coefficient is identified:

(1) $$\begin{array}{c} \psi =\frac{A_{H}}{A_{S}}=\frac{\int_{0}^{q1}F_{OB}\left(q\right)dq-\int_{0}^{q1}F_{O\;D}\left(q\right)dq}{\int_{0}^{q1}F_{S}\left(q\right)dq}, \end{array}$$

where *A*_*H*_ is the surface area of the hysteresis loop, *A*_*S*_ is the surface area under the curve representing elastic properties of the disc, *F*_*OB*_ (*q*) is the load curve equation, *F*_*OD*_ (*q*) is the load curve equation, and *F*_*S*_ (*q*) is the curve equation representing elastic properties of the TMJ disc.

(2) $$\begin{array}{c} F_{OB}\left(q\right)=a_{1}\cdot q+a_{2}\cdot q^{2}+a_{3}\cdot q^{3},\\ F_{O\;D}\left(q\right)=b_{1}\cdot q+b_{2}\cdot q^{2}+b_{3}\cdot q^{3}+b_{4}\cdot q^{4}+b_{5}\cdot q^{5}. \end{array}$$

Open representations of load and unload curves constitute the basis for setting static characteristics of the joint disc, which is calculated as an arithmetic mean. Additionally, static characteristics of discs may be approximated with great accuracy by a polynomial function of the third degree:

(3) $$\begin{array}{c} F_{S}\left(q\right)=\frac{F_{OB}\left(q\right)+F_{O\;D}\left(q\right)}{2}=d_{1}\cdot q+d_{2}\cdot q^{3}. \end{array}$$

The methodology presented in this chapter is a formal basis for identification of biomechanical properties of discs tissues. Based on physically identified parameters, it is possible to thoroughly reconstruct the phenomena occurring in the discs, using the nonlinear rheological model Kelvin–Voigt.

**Figure 3:**
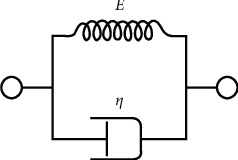
Kelvin–Voigt model-schematic representation.

A Kelvin–Voigt material, also called a Voigt material, is a viscoelastic material having the properties both of elasticity and viscosity. The Kelvin–Voigt model, also called the Voigt model, can be represented by a purely viscous damper and purely elastic spring connected in parallel, as shown in Figure 3.

Since the two components of the model are arranged in parallel, the strains in each component are identical:

(4) $$\begin{array}{c} \varepsilon_{\text{Total}}=\;\varepsilon_{S\;}=\;\varepsilon_{D\;}, \end{array}$$

where the subscript *D* indicates the stress-strain in the damper and the subscript *S* indicates the stress-strain in the spring. Similarly, the total stress will be the sum of the stress in each component:

(5) $$\begin{array}{c} \sigma_{\text{Total}}=\;\sigma_{S}+\;\sigma_{D}. \end{array}$$

From these equations, we get that, in a Kelvin–Voigt material, stress *σ*, strain *ε*, and their rates of change in time *t* are governed. The nonlinear rheological model proposed in this paper, which was used to model the articular disc tissues, is a good example of an experiment carried out on a testing machine.

## 3. Results

One of the fundamental elements of the model tests is the identification of physical parameters, which are stiffness and the energy dispersion coefficient. Biomechanical properties of evaluated discs, in particular the ability to recover the primary shape, after cessation of the load, are an individually variable feature. Under load conditions, both static and dynamic, in biological tissues, there are rheological phenomena, as a result of which the processes occurring in biological materials are predominantly reversible. In contrast to construction materials (technical), plastic deformation is most often of permanent nature. Despite the differences existing between biological and construction materials, the methodology of identification proceeds similarly, as the phenomenological description of phenomena is similar. In the identification of discs, biomechanical properties are carried out in the same manner as in the case of technical materials, such as rubber or steel. Means and standard deviations of biophysical parameters typical of dissipation properties of the studied TMJ discs in subsequent load cycles are specified in Table 1.

**Figure 4:**
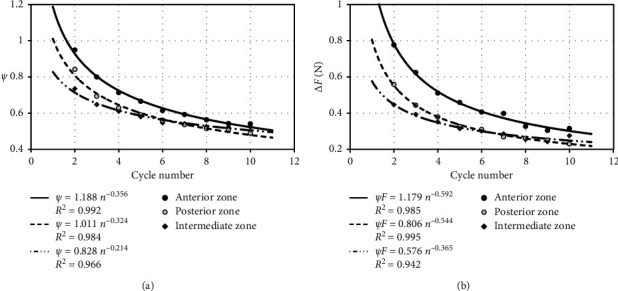
Average values calculated for particular load cycles: (a) energy dispersion coefficient *ψ*; (b) parameter Δ*F*.

**Figure 5:**
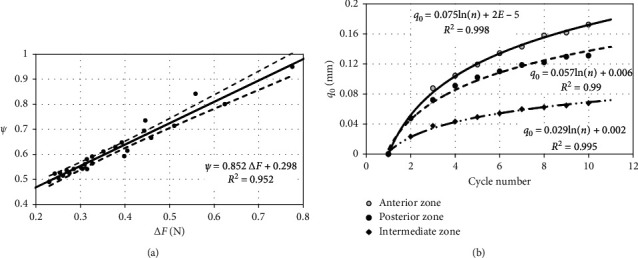
Model test results illustrating the effect of (a) energy dispersion coefficient *ψ* and (b) number of cycles on the parameter *q*_0_.

**Figure 6:**
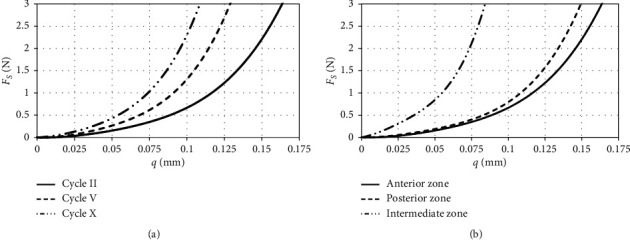
Course of static characteristics depending on (a) number of loading cycles and (b) TMJ disc load area.

The effect of cyclical load on biomechanical properties of particular TMJ disc zones is shown graphically in Figures 4–6.

With the next load cycle, the disc tissue loses its ability to dissipate energy. In general, the dissipating properties are treated as a natural shock absorber, protecting the TMJ against overload (Figure 3).

The number of load cycles affecting disc tissues influences similarly parameter Δ*F* as the number of load cycles concerning the energy dispersion coefficient *ψ* (Figure 4(b)). The most significant difference of forces was observed during cyclical disc loading in the AZ area, while the lowest in the IZ zone. Between parameter Δ*F* and energy dispersion coefficient *ψ*, there is a linear causal relation (Figure 5(a)). The above dependence has been determined based on averaged data included in Table 1. This relation enables conducting an initial assessment of dissipation properties of discs already at the stage of experimental research. For this purpose, based on the prepared force-displacement characteristics, the value of parameter Δ*F* in the recorded load cycles should be determined. The upper and the lower confidence intervals pictured in the chart were calculated assuming the level of significance *α* = 0.01. Based on completed measurements on the testing machine, it was stated that, after reaching a specified number of cycles, the material from which the discs are built reaches saturation. This phenomenon can be recorded on force-displacement characteristics and manifests itself in the stabilization of the hysteresis loop. One of the methods ensuring identification of the number of cycles at which disc tissue achieves saturation is the determination of initial displacements *q*_0_ (Figure 5(b)). In the discussed approach, the disc has achieved saturation at the time of stabilization of the characteristics. The determined approximating curves clearly indicate that disc tissues achieve saturation most rapidly in the IZ zone. Regardless of the zone of disc load with external force, its static characteristics FS is stiffened along with the increase in the number of cycles (Figure 6(a)). The increment in load cycles makes elastic properties of examined areas show similar properties (Figure 6(b)). Additionally, primarily, it comes to levelling the rigidity of AZ and PZ disc regions, and it is determined by the thickness of particular examined areas.

The analysis of the obtained results indicates that the identified biomechanical properties of TMJ disc tissues may be with high accuracy, approximated with functions: polynomial, power, and logarithmic.

The model tests carried out indicate that, with the cyclic loading, the stiffness of the disc tissues increases (Figure 6). An increase in stiffness in individual load cycles causes a change in the directional factor of the biomechanical characteristics reproducing the relationship between displacement *q* and force F. The biomechanical characteristics in Figure 6 indicate that the stiffness of the disc in AZ and PZ zones is comparable. In the IZ zone, on the contrary, the disc is almost twice as rigid.

The change in biomechanical properties in particular cycles was identified based on hysteresis loops, which corresponded to particular load cycles. The loss of energy dissipation capacity is directly related to the reduction of hysteresis loops. In other words, the smaller the area of the hysteresis loop, the lower the energy dissipation capacity of the joint disc.

## 4. Discussion

TMD etiology has been proven to be multifactorial, and bite disorders may be one of the causes. The symptoms of TMD correlate with age, sex, and dental and occlusal conditions. [3, 4, 40]. The occurrence of bone lesions in the condyles correlates with age but poorly correlates with gender and condition of teeth and occlusion in patients with and without TMD [41].

In the correct occlusion, while clenching strong teeth, TMJ is not subject to greater loads. On the contrary, when it comes to the emergence of premature contacts, then it is possible to observe growth in occlusal forces. In normal occlusion, TMJ is generally not subject to greater strain when clamping teeth. In some patients, an increase in occlusive forces can be observed in case of premature contacts and increased chewing muscle tension.

These disorders may be natural and may also result from the actions of dentists. Both can lead to unnatural chewing patterns. This abnormal chewing pattern occurs in unnatural positions within the TMJs and the articular discs, resulting in dislocation, joint sounds, and pain in and around the TMJs. With age, there are also changes in condylar morphology: the condylar head and joint nodule are flattened. No changes in the joint angle (CPI) are observed [41]. Increased muscle tension also occurs in patients with bruxism. Few studies survey the relationship between bruxism and occlusal interferences. Manfredini et al. show that significant factors in the development of occlusal parafunctions are malocclusions and abnormal bites. It should be stressed that an occlusal aspect most often connected with psychological disorders gives the picture of full-blown bruxism [42].

The researcher also examined the psychic and occlusal factors in bruxism and concluded that there is an association between balancing side interferences and bruxism [42]. The stomatognathic system of patients appears to be able to accept and adapt to occlusal alterations because it has considerable adaptability. However, adaptation is possible within certain biological limits individually for each patient. Despite the substantial number of published papers about “TMD” and “occlusion,” there are still controversy and contradictory opinions on the interaction between “occlusion” and “temporomandibular disorders.” There is still existing confusion and contradiction in the dental literature about the role of “occlusion” in “TMD” [43].

It should also be borne is mind that the injuries as well as pathological states of the mastication organ may have an adverse impact on the biomechanical properties of discs. The damage created during injuries may often exceed the regenerative possibilities of TMJ discs and articular surfaces. The continuous, long-term load may be a source of increased friction, occurring between the disc and articular areas [44, 45]. This kind of load often contributes to an increase in porosity of articular surfaces. Excessive friction and load exceeding the maximum strength of the discs lead directly to the perforation of disc tissues. Tensile strength of disc tissues depends mainly on the direction and the area of external load. For instance, as Beatty and coauthors state [22], the maximum strength of IZ disc area is 37.4 MPa, when tensile stress operates in the direction front-back. While the load affects the central-transverse direction, the maximum strength is ca. 1.6 MPa.

The largest number of information about biomechanical properties of discs was obtained from research conducted on pigs and bovine discs, as they are structurally and functionally similar to human discs, which is indicated by some authors: Bermejo et al. [46] and Gonzales et al. [47]. Anatomically, in both of them, it is possible to distinguish strengthened anterior and posterior areas. In contrast, the IZ area is much thinner and hence more susceptible to the adverse effects of the load. The results of the assessments of the degree of human and pigs discs similarity are presented in the publication by Chladek and Czerwik [48], in which their thickness was measured down to 0.01 mm and the size of penetrator spherical cavity with a diameter of 5 mm. Additionally, the penetrator was pressed into the discs for ca. 15 s with force equal to about 1.2 N. As the similarity criterion the researchers assumed the percentage size of penetrator's cavity referred to the thickness of discs. Average relative errors of the thickness of human and pig discs in the AZ area amounted to ca. 7%, IZ ca. 31%, and PZ ca. 27%. On the contrary, average relative errors estimated based on penetrator's cavity amounted to 29% in the AZ area, 2% in the IZ area, and 27% in the PZ area. Such a similarity is sufficient and acceptable for implementation of further research, in the scope of the model loads evaluation, operating on human TMJ.

Research concerning the assessment of biomechanical properties of discs has already been published in many studies, e.g., Tanne et al. [49], Chin et al. [50], Lai et al. [51], and Tanaka et al. [52–54]. Comparing, however, research results obtained from various experiments causes tremendous difficulties, first of all, because discs are characterized by a nonuniform internal structure and individually variable biophysical properties. Also, the different course of the performed experiments is essential. The diversified methodology of conducting experimental research determines a broad spread of identified values of the elasticity modules that is within 1 to 100 MPa. Besides, its value is significantly affected by the speed of the studied tissue load. According to Beatty [22], the elasticity module of the pig disc stretched at 0.5 mm/s is ca. 27 MPa.

On the contrary, the elasticity module of the disc stretched at 500 mm/s is over three times greater and is ca. 83 MPa. These data indicate that it is necessary to define the guidelines that would standardize the course of experiments conducted on biological tissues. Only ensuring appropriate standards will enable us to compare the results obtained by various researchers. In addition, the main question should be asked: is the description of the elastic properties of discs showing nonlinear characteristics with Young's elasticity modulus reasonable methodically? Young's elasticity modulus is the coefficient including relations occurring between the stress and deformation following Hooke's law that characterizes mechanical properties of the materials only when they undergo elastic deformation. In a general sense, Young's elasticity modulus defines the directional coefficient of characteristic's inclination. In the case of materials showing linear mechanical properties, Young's modulus accepts a constant value regardless of material deformation. However, for materials of a nonlinear stress-deformation characteristics course, it is possible to determine many elasticity modulus values. It is caused by the fact that depending on the material deformation value, the directional coefficient of the tangential to characteristics is changed. Therefore, we are confronted by such a large spread of Young's modulus value, which can be found in published works.

Specialist literature, apart from publications where the authors focused on the evaluation of Young's elasticity modulus, on both human and animal discs, is also devoted to forcing-displacement characteristics research and stress-deformation. An example of this type of research is results published in the work by Beek et al. [55, 56] that were conducted on human discs from donors aged 73 to 86. On a specially designed laboratory post, the discs were deformed in cycles with a sinusoidal course in different areas. The obtained results indicate that, regardless of the deformation area, peak stress decreases with every subsequent cycle to the level until an agreed condition is reached. Additionally, peak stress in principle was determined after the fifth cycle. Such a nature of the course of biomechanical phenomena indicates cyclic relaxation of disc tissues. At this point, one should be reminded that cyclical material deformation in practice comes down to controlling cross-beam movement of the testing machine.

On the contrary, cyclical load differs from cyclical deformation because during material loading, the size controlled by the system of automatic regulation of the testing machine is the force. Results of identification presented in this paper were obtained in cycles loading discs, as a result of which the recorded temporary courses of deformation show the characteristics of cyclical crawling. From a theoretical point of view, the way of affecting material tissue plays no significant role, since, during cyclical deformation, as well as disc tissues load, part of the biomechanical energy is dispersed, as is stated by Tanaka and Van Eijden [57]. The formal basis for identifying its dissipation and elastic properties is hysteresis loops that are built based on force and displacement signals.

Based on the results of experimental and model tests, it can be stated that the number of load cycles substantially affects the surface area of the hysteresis loop, which means that, regardless of the zone of the examined disc area, its dissipation properties are being limited (Figure 3(a)).

A comparison of prepared dissipation properties of disc in AZ, IZ, and PZ zones indicates that the lowest ability to dissipate energy can be observed in the IZ disc zone. Accordingly, high number of load cycles, above five, makes the dissipation properties of particular disc zones demonstrate similar properties. The phenomenon taking place inside pig disc tissues is also proven by an increase in deformation, progressing despite unloading (Figure 2(b)). This phenomenon is visible very clearly in the first load cycles. This effect is limited along with subsequent cycles, and it should be assigned to the phenomenon of strengthening. Bearing the above in mind, it was decided to determine, as a significant function, also in each load cycle parameter *ΔF*, representing the difference of forces recorded during the loading and unloading of the TMJ disc. Additionally, this difference can be found for displacement *q*_1_ defining the maximum value of recorded force during the loading phase. It is worth mentioning the fact that this phenomenon also exists in human disc tissues, which is proven by experimental research published in the work by Beek et al. [56].

We should have in mind that the biomechanical properties of discs are subject to changes as a result of various life factors. Comprehensive research was conducted as to alteration of viscoelastic properties, caused by ageing of the body, in skin or ligaments [58]. For instance, the elasticity modulus of a rat's skin increases along with maturation and decreases with the ageing of the body [59]. On the contrary, the authors in [60] demonstrated that, with the ageing of the body, the calcium content in discs increases. Lai, along with collaborators [51], showed that the elasticity module of human discs grew with age and suggested that it may be related to lowering the capability of collagen to remodel. Apart from collagen fibres, the disc contains a small number of flexible fibres. Collagen fibres provide the shape of the discs, while flexible ones are responsible for the restoration of its primary shape after cessation of load [61]. Collagen fibres are usually folded, at the moment of stretching the disc in the first place, and folds are stretched [62, 63]; however, in this phase, collagen fibres do not carry load [64]. Only in the subsequent phase, collagen fibres are stretched, and hence, they begin to carry the load.

Additionally, initially, these loads are small since the collagen net impedes the flow of interstitial fluid [65]. Further load makes the collagen net deform, as a result of which it comes to pressing the interstitial fluid from the loaded place through pores in the collagen net. The flow of fluid, as well as the change in the placement of collagen fibres, is reversible until the moment when the disc is deformed beyond the scope of physiological deformations. On the basis of the presented information, it can be assumed that, during the identification of biomechanical properties of disc tissues, it is inadvisable to conduct tests on cut-out samples. That is because during loading the cut samples, interstitial fluid is pressed out, as a result of which overestimated rigidity values are obtained. The effect of the preparation of samples for stress-deformation characteristics was presented in the work by Chladek and Czerwik [48]. Bearing in mind the information contained there, in this paper, we conducted experimental research only on discs, whose tissue consistency was not disturbed.

The authors of the manuscript are aware that despite its many advantages, the presented studies are not without limitations. They included a small number of tests and were carried out in conditions that are significantly different from physiological ones. However, as already mentioned, the movements of condylar are so complex that they cannot be simulated outside the living organism and, hence, the model tests.

## 5. Conclusions

The obtained results indicate that the cyclical loads affecting the joints adversely affect the ability of discs to dissipate energy:

1. The most significant ability to dissipate energy was observed in the anterior and posterior zones of the TMJ disc. In the intermediate zone, the energy dissipation capacity is approx. 20% less than the anterior zone and about 10% less than the posterior zone.
2. The joint disc tissues lose their ability to dissipate energy from the first load cycle. After ten load cycles, the energy dissipation capacity decreased by about 40%.
3. Concerning the elastic properties, the intermediate zone is twice as rigid as the anterior and posterior zones. After ten load cycles, a 30% increase in joint disc stiffness was observed.

Such a statement is supported by clinical cases concerning patients with symptoms of teeth gritting and teeth occluding and excessively chewing gum, in whom symptoms of the mastication organ dysfunctions are observed. The obtained results indicate the importance of relaxation of masticatory muscles and unloading of the articular structures, both in the prevention and the treatment of temporomandibular conditions.

The results show how important it is to relax the masticatory muscles and relieve the stress on the joint structures, both in the prevention and treatment of TMD.

## Figures and Tables

**Table 1:** Dissipation properties of transport discs.

Cycle no.	Anterior zone		Intermediate zone		Posterior zone	
	*ψ*	∆*F* (N)	*ψ*	∆*F* (N)	*ψ*	Δ*F* (N)
	Mean (SD)	Mean (SD)	Mean (SD)	Mean (SD)	Mean (SD)	Mean (SD)
2	0.94 (0.27)	0.78 (0.09)	0.73 (0.15)	0.45 (0.18)	0.84 (0.27)	0.56 (0.46)
3	0.80 (0.22)	0.62 (0.10)	0.65 (0.08)	0.39 (0.11)	0.69 (0.23)	0.44 (0.38)
4	0.71 (0.18)	0.51 (0.08)	0.61 (0.07)	0.35 (0.11)	0.63 (0.20)	0.38 (0.31)
5	0.67 (0.17)	0.46 (0.07)	0.58 (0.07)	0.31 (0.12)	0.59 (0.16)	0.33 (0.24)
6	0.61 (0.15)	0.41 (0.06)	0.55 (0.07)	0.30 (0.13)	0.56 (0.15)	0.31 (0.24)
7	0.59 (0.14)	0.40 (0.05)	0.54 (0.07)	0.29 (0.11)	0.54 (0.13)	0.27 (0.20)
8	0.56 (0.13)	0.33 (0.04)	0.53 (0.07)	0.25 (0.10)	0.51 (0.12)	0.26 (0.18)
9	0.54 (0.12)	0.30 (0.04)	0.52 (0.07)	0.24 (0.10)	0.51 (0.11)	0.25 (0.15)
10	0.54 (0.12)	0.32 (0.02)	0.52 (0.08)	0.28 (0.12)	0.49 (0.10)	0.23 (0.14)

## Data Availability

The data used to support the findings of this study are available from the corresponding author upon request (edward.kijak@umed.wroc.pl).
